# *Hirsutella sinensis* mycelium suppresses interleukin-1β and interleukin-18 secretion by inhibiting both canonical and non-canonical inflammasomes

**DOI:** 10.1038/srep01374

**Published:** 2013-03-05

**Authors:** Tsung-Teng Huang, Kowit-Yu Chong, David M. Ojcius, Yi-Hui Wu, Yun-Fei Ko, Cheng-Yeu Wu, Jan Martel, Chia-Chen Lu, Hsin-Chih Lai, John D. Young

**Affiliations:** 1 Center for Molecular and Clinical Immunology, Chang Gung University, Taoyuan, Taiwan, Republic of China; 2 Department of Medical Biotechnology and Laboratory Sciences, Chang Gung University, Taoyuan, Taiwan, Republic of China; 3 Laboratory of Nanomaterials, Taoyuan, Chang Gung University, Taiwan, Republic of China; 4 Research Center of Bacterial Pathogenesis, Chang Gung University, Taoyuan, Taiwan, Republic of China; 5 Molecular Cell Biology, Health Sciences Research Institute, University of California, Merced, Merced, California, United States of America; 6 Cancer Research Center, National Cheng Kung University Hospital, Tainan, Taiwan, Republic of China; 7 Chang Gung Biotechnology Corporation, Taipei, Taiwan, Republic of China; 8 Biochemical Engineering Research Center, Ming Chi University of Technology, Taipei, Taiwan, Republic of China; 9 Department of Respiratory Therapy, Fu Jen Catholic University, Taipei, Taiwan, Republic of China; 10 Laboratory of Cellular Physiology and Immunology, Rockefeller University, New York, New York, United States of America

## Abstract

*Cordyceps sinensis* is a medicinal mushroom used for centuries in Asian countries as a health supplement and tonic. *Hirsutella sinensis*—the anamorphic, mycelial form of *C. sinensis*—possesses similar properties, and is increasingly used as a health supplement. Recently, *C. sinensis* extracts were shown to inhibit the production of the pro-inflammatory cytokine IL-1β in lipopolysaccharide-treated macrophages. However, the molecular mechanism underlying this process has remained unclear. In addition, whether *H. sinensis* mycelium (HSM) extracts also inhibit the production of IL-1β has not been investigated. In the present study, the HSM extract suppresses IL-1β and IL-18 secretion, and ATP-induced activation of caspase-1. Notably, we observed that HSM not only reduced expression of the inflammasome component NLRP1 and the P2X_7_R but also reduced the activation of caspase-4, and ATP-induced ROS production. These findings reveal that the HSM extract has anti-inflammatory properties attributed to its ability to inhibit both canonical and non-canonical inflammasomes.

Medicinal mushrooms have been used for centuries in Asia as folk medicine and natural health tonics^1^,^2^. Mushrooms like *Cordyceps sinensis*, *Ganoderma lucidum*, and *Agaricus blazei* Murrill have been used for various human conditions, including autoimmune disease, cancer, chronic inflammation, fatigue, and type II diabetes. Recent research has shown that these mushrooms produce antiviral, anticancer, anti-inflammatory, and immunomodulatory effects on cultured cells and laboratory animals. Current research efforts are directed towards identifying the compounds responsible for mediating these biological effects, with polysaccharides and nucleosides appearing as major candidates^1^,^2^.

*C. sinensis* (also termed *Ophiocordyceps sinensis*) is an ascomycete fungus that possesses a peculiar mode of growth characterized by two main stages; the first stage is characterized by the fungus infecting underground caterpillar larvae in the winter, whereas the second stage is associated with the production of a fruiting body that protrudes from the dead caterpillar's head and grows above the ground during the summer^3^,^4^,^5^,^6^,^7^. For this reason, *C. sinensis* is known as the “caterpillar fungus” or “dong-chong-xia-cao” (literally “winter worm, summer grass” in Chinese)^6^,^7^,^8^. The growth of this natural fungus is also unusual due to the fact that it is limited to the Tibetan plateau and southwestern China, and it usually grows at or even below the relatively low temperature of 18°C^4^,^5^,^9^. Recent studies indicate that *C. sinensis* has a wide range of biological activities, including anti-tumor^10^,^11^, immunomodulatory^12^,^13^, anti-inflammatory^14^,^15^, anti-oxidant^16^,^17^, anti-infection^18^, and anti-aging properties^19^.

Due to the rarity of natural *C. sinensis*, other means of producing this fungus have been investigated. The identity of the anamorph of *C. sinensis* has been a topic of considerable controversy in the past^20^. *Hirsutella sinensis*, which today is widely accepted as the true anamorphic, mycelial stage of natural *C. sinensis*^20^, is amenable to culture in vitro, and is increasingly used as a health supplement. Studies of the pharmacological properties of HSM have shown that it possesses biological activities similar to that of the wild mushroom. For instance, these activities include reduction of drug-induced leucopenia following kidney transplantation, amelioration of radiation-induced toxicity, and stimulation of immune cells in vivo^21^,^22^. Earlier, we demonstrated that HSM prolongs survival and decreases symptom severity in a murine model of the systemic autoimmune disease, lupus erythematosus^23^. However, the mechanism underlying the immunosuppressive effects of HSM is still unclear.

Methanol extracts of natural *C. sinensis* have been shown to suppress bronchoalveolar lavage fluid (BALF) cell proliferation and to reduce IL-1β, IL-6, IL-8, IL-10 and tumor necrosis factor (TNF)-α production in LPS-activated BALF cell cultures^24^. Li et al. reported that *C. sinensis* water extracts reduce the production of the pro-inflammatory cytokines IL-1β, IL-6, TNF-α, and IL-12p70 in LPS-activated dendritic cells^25^. Nonetheless, whether HSM possesses similar activities has not been investigated.

Macrophages are differentiated immune cells that originate as blood monocytes and are found in tissues throughout the body. These immune cells play an essential role during initiation and propagation of inflammatory responses by producing pro-inflammatory cytokines such as IL-1β, IL-18, and TNF-α, as well as other inflammatory mediators like nitric oxide and prostaglandins^26^,^27^,^28^. IL-1β and IL-18, which are members of the IL-1 cytokine superfamily, promote a variety of innate immune processes associated with infection, inflammation, and autoimmunity^29^,^30^. IL-1β participates in the generation of systemic and local immune responses against various strains of pathogens, and has been implicated in the pathogenesis of inflammatory diseases, such as gout, asthma, inflammatory bowel diseases, rheumatoid arthritis, and atherosclerosis^31^,^32^,^33^. IL-18 also plays a critical role in the execution of anti-microbial and anti-viral immunity, and this cytokine has been associated with severe inflammatory disorders, such as rheumatoid arthritis, Crohn's disease, psoriasis, lupus, sarcoidosis, and multiple sclerosis^34^,^35^.

The pro-inflammatory cytokines, IL-1β and IL-18, are synthesized as inactive precursors (i.e., pro-IL-1β and pro-IL-18) and accumulate within the cytosolic compartment of monocytes and macrophages exposed to or “primed” with pathogen-associated molecular patterns (PAMPs) like the bacterial endotoxin LPS^36^. However, LPS by itself is usually insufficient to trigger IL-1β and IL-18 secretion from macrophages unless danger-associated molecular patterns (DAMPs) provide the second signal responsible for the activation of the inflammasome complex, activation of the protease caspase-1, processing of pro-IL-1β and pro-IL-18, and release of the mature cytokines from the cells^37^,^38^,^39^.

Extracellular adenosine 5′-triphosphate (ATP) acts as a danger signal released from injured cells during tissue damage and inflammation; it initiates inflammation and further amplifies and sustains cell-mediated immunity through P2 receptor-mediated purinergic signaling^40^,^41^. Binding of ATP to the P2X_7_ receptor (P2X_7_R) in primed monocytes and macrophages leads to inflammasome activation and secretion of pro-inflammatory cytokines IL-1β and IL-18^42^.

Inflammasomes represent a group of cytoplasmic multiprotein complexes whose assembly leads to activation of the cysteine protease caspase-1, which promotes the proteolytic processing of the immature forms of IL-1β and IL-18^43^. The inflammasome complex is typically formed by three components consisting of a nucleotide binding and oligomerization domain (NOD)-like receptor (NLR), the ASC adaptor protein (for apoptosis-associated speck-like protein containing a caspase recruitment domain), and pro-caspase-1. Upon activation, oligomerized NLRs interact with ASC, which in turn recruits and activates caspase-1 and leads to cleavage and activation of pro-IL-1β and pro-IL-18^39^. The NLRP1 (nacht, leucine-rich repeat and pyrin domain containing domain-1; also known as NALP1, NAC, CARD7, DEFCAP, or CLR17.1) and NLRP3 (also known as NALP3, cryopyrin, CIAS1, or PYPAF1) inflammasomes are two of the best-characterized canonical inflammasomes described so far. A large number of stimuli have been shown to trigger activation of the NLRP3 inflammasome, including ATP, monosodium urate crystals, cholesterol crystals, UVB irradiation, pathogen-derived nucleic acids, silica, asbestos, and amyloid-β^44^,^45^,^46^,^47^,^48^,^49^,^50^,^51^. LPS and muramyl dipeptide (MDP) along with ATP have been reported to induce NLRP1 inflammasome assembly, caspase-1 activation and cleavage of pro-IL-1β into its active form^52^,^53^.

More recently, non-canonical inflammasomes containing murine caspase-11 have also been reported^54^,^55^. Caspase-11 does not exist in humans, but is functionally equivalent to caspase-4 and caspase-5, which also modulate inflammasome activity^56^,^57^.

*C. sinensis* extracts were previously shown to inhibit the production of IL-1β in LPS-stimulated macrophages. However, the molecular mechanism responsible for this inhibition was not characterized, and the possibility that IL-18 secretion may also be affected was not investigated. The main objective of the present study was to determine whether ethanol extracts of HSM have an inhibitory effect on the production of IL-1β and IL-18 in LPS-primed human macrophages. In addition, we examined whether the HSM ethanol extract can modulate inflammasome activation in macrophages. We demonstrate that HSM ethanol extract suppresses IL-1β and IL-18 secretion. The reduction of IL-1β and IL-18 production is associated with down-regulation of NLRP1, a component of one of the canonical inflammasomes. HSM also inhibits the transcription and activation of both caspase-1 and caspase-4, the latter being associated with non-canonical inflammasomes. Furthermore, ATP-induced ROS generation and P2X_7_R activation are suppressed by HSM.

## Results

### Absence of toxicity of the HSM ethanol extract on human macrophages

**Figure 1:**
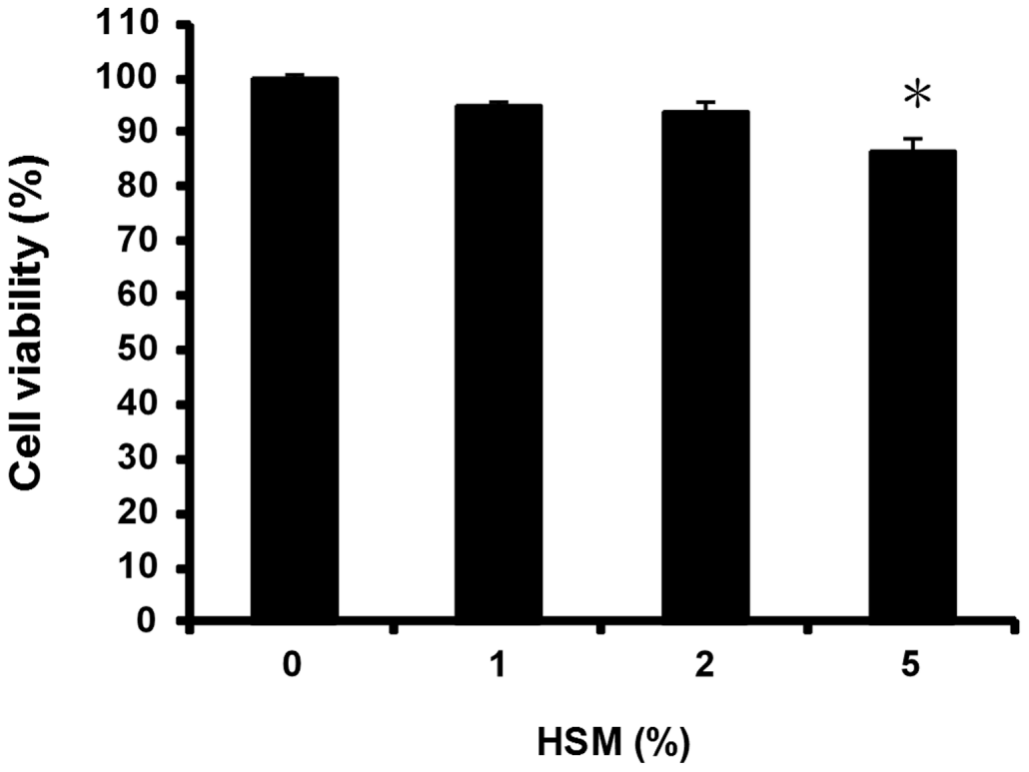
Absence of toxicity due to HSM treatment of human macrophages. Cells were treated with 1 to 5% of HSM ethanol extract for 24 h, and cell viability was measured by the MTT assay, as described in Materials and Methods. Data are presented as means ± SE of three experiments preformed in duplicate. *P < 0.01 versus HSM-untreated control cells.

Whether ethanol extracts of HSM have cytotoxic effects on human cells has not been studied. Therefore, we first determined the effects of the HSM extract on the viability of THP-1 macrophages using the MTT assay. Treatment of the cells with either 1 or 2% (v/v) of the HSM ethanol extract for 24 h did not affect cell viability, compared with HSM-untreated control cells (Figure 1). However, cell viability was significantly decreased when the cells were incubated with 5% of HSM ethanol extract for the same period of time (Figure 1). Based on these results, we used the HSM extract at a concentration of 1 or 2% in subsequent experiments.

### HSM extract reduces ATP-induced IL-1β and IL-18 secretion in LPS-primed macrophages

**Figure 2:**
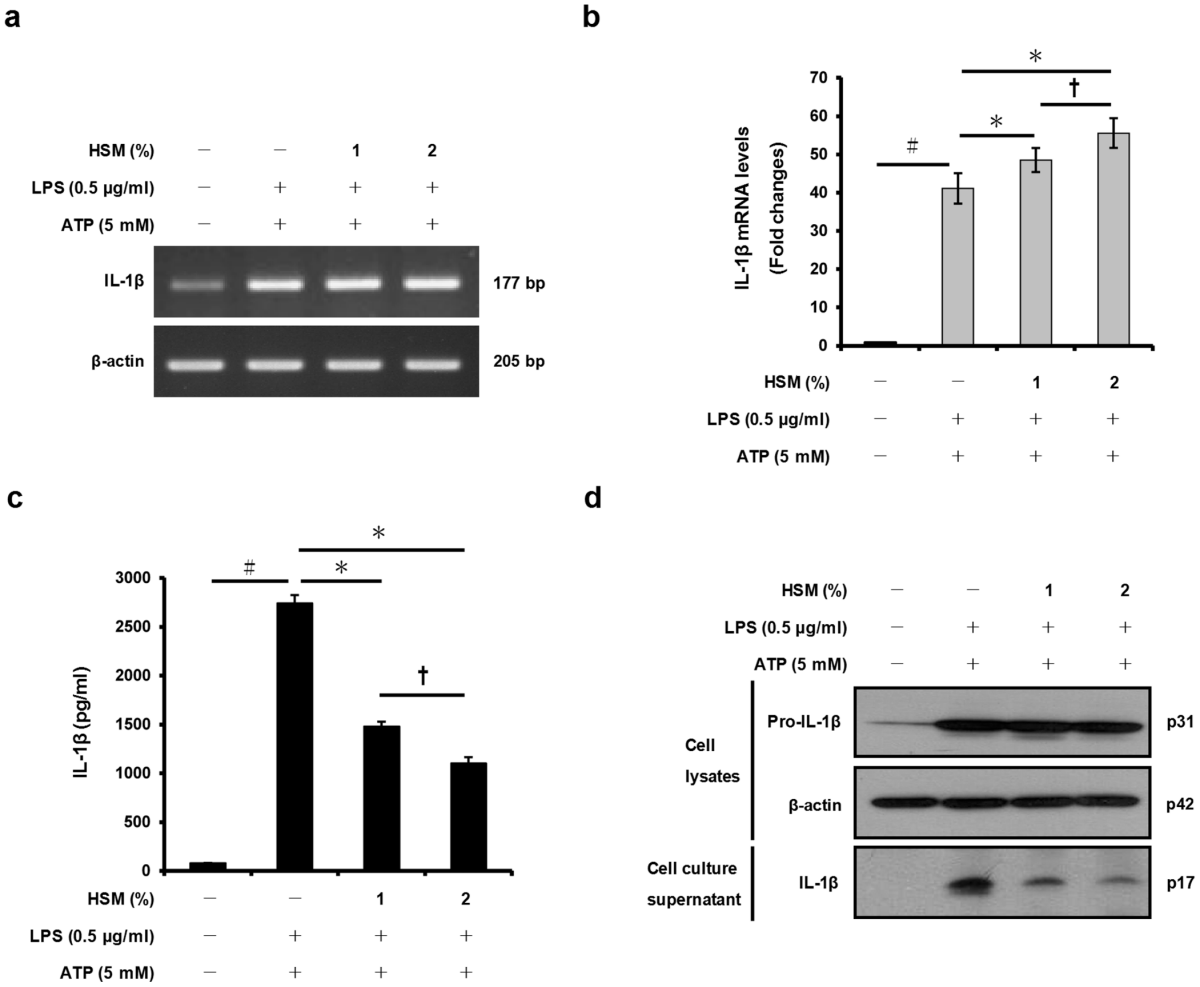
Effects of HSM on IL-1β gene expression and secretion in human macrophages. Cells were pre-treated with either 1 or 2% of HSM extract for 20 h, followed by treatment with LPS (0.5 μg/ml) for 3 h and with ATP (5 mM) for 1 h. (a) The mRNA expression levels of IL-1β were determined by RT-PCR analysis. (b) IL-1β mRNAs were quantified using real-time PCR. β-actin gene expression was used for normalization. The results are expressed as fold changes, considering one as the value of untreated cells. (c) The amount of IL-1β in cell culture supernatants was detected by ELISA. (d) The presence of IL-1β in cell lysates and cell culture supernatants were analyzed by Western blot analysis. Data are presented as means ± SE of three experiments performed in duplicate. ^#^*P* < 0.01 versus untreated cells. *P < 0.01 versus HSM-untreated (ethanol-treated) control cells. **^†^***P* < 0.05 versus HSM (1%) treated cells.

**Figure 3:**
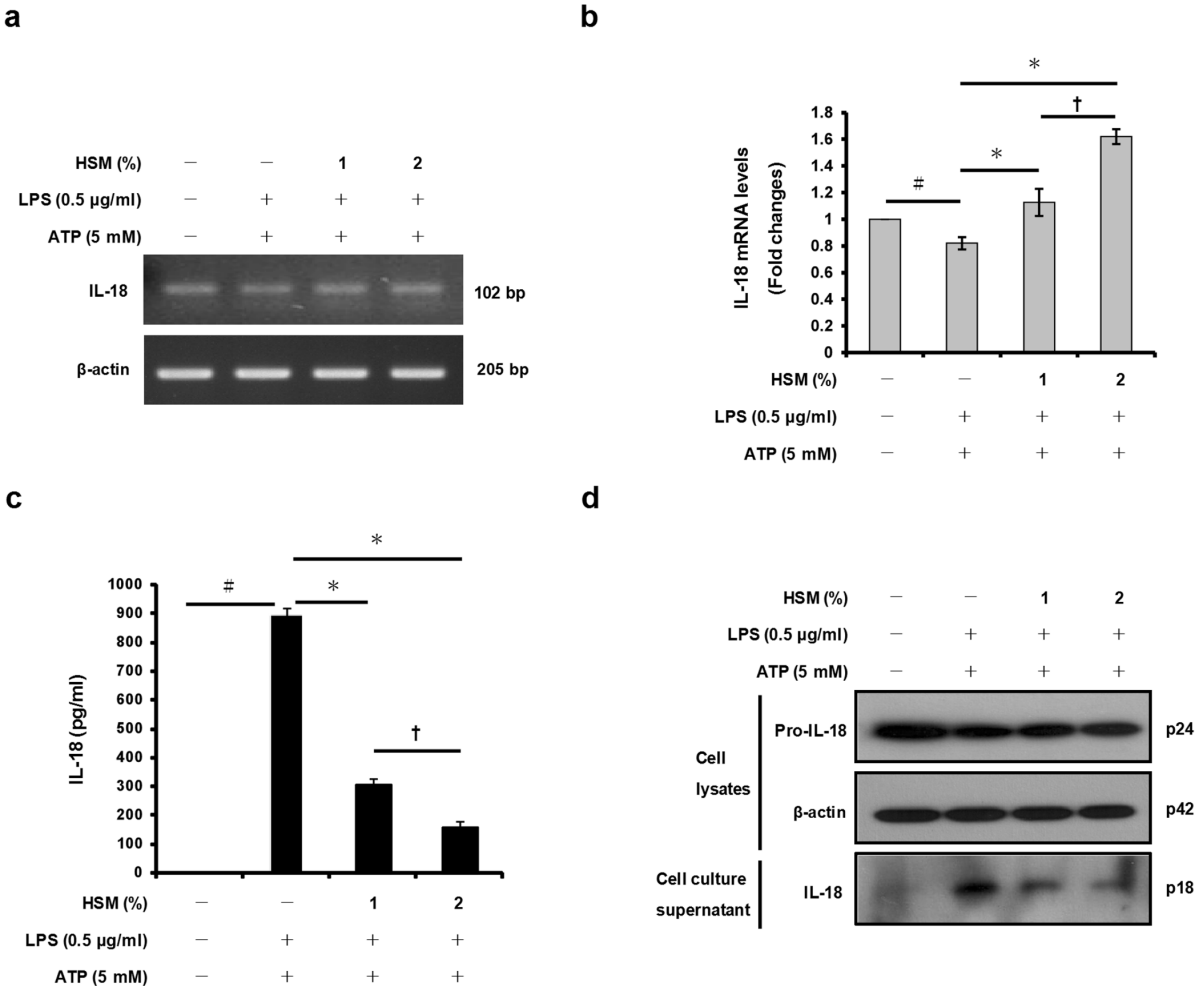
Effects of HSM on IL-18 gene expression and secretion in human macrophages. Cells were pre-treated with various concentrations (1 or 2%) of HSM extract for 20 h, followed by treatment with LPS (0.5 μg/ml) for 3 h and ATP (5 mM) for 1 h. (a) The mRNA expression levels of IL-18 were determined by RT-PCR analysis. (b) IL-18 mRNAs were quantified using real-time PCR. β-actin gene expression was used for normalization. The results are expressed as fold changes, considering one as the value of untreated cells. (c) The amount of IL-18 in cell culture supernatants was detected by ELISA. (d) The presence of IL-18 in cell lysates and cell culture supernatants were analyzed by Western blot analysis. Data are presented as means ± SE of three experiments performed in duplicate. ^#^*P* < 0.01 versus untreated cells. *P < 0.01 versus HSM-untreated control (ethanol) cells. **^†^***P* < 0.05 versus HSM (1%) treated cells.

We examined whether the HSM extract affects IL-1β and IL-18 gene expression in THP-1 macrophages. We first pre-treated the macrophages with the HSM ethanol extract (1 or 2%) for 20 h, then with LPS (0.5 μg/ml) for 3 h to induce cytokine expression, and finally with ATP (5 mM) for 1 h to activate the cells and induce secretion of IL-1β and IL-18. RT-PCR and quantitative real-time PCR analyses showed that HSM pre-treatment increased the mRNA expression levels of IL-1β and IL-18 in a dose-dependent manner (Figure 2a, 2b, 3c, and 3d).

We then determined the concentrations of secreted IL-1β and IL-18 proteins in the cell culture supernatants of the same ATP-activated macrophages. ELISA and Western blot analyses revealed that pre-treatment of the cells with HSM significantly reduced the secretion of IL-1β and IL-18 in a dose-dependent manner (Figure 2c, 2d, 3c, and 3d). These findings indicate that the HSM extract stimulates expression of the cytokines, but decreases their secretion in response to ATP treatment.

### HSM extract suppresses ATP-induced caspase-1 activation in macrophages

**Figure 4:**
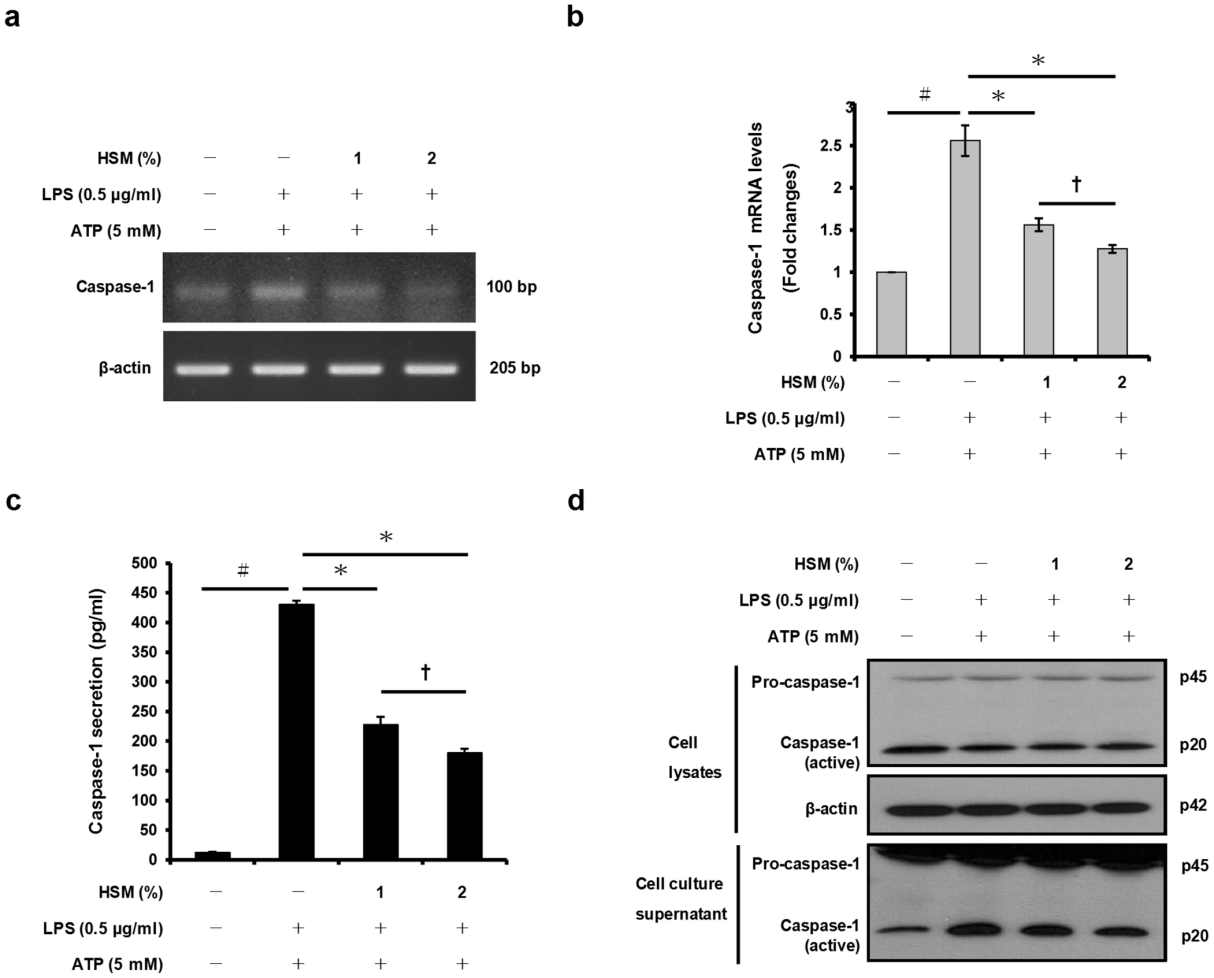
Effects of HSM on ATP-mediated caspase-1 gene expression and activation in human macrophages. Cells were pretreated with HSM extracts (1 or 2%) for 20 h, followed by treatment with LPS (0.5 μg/ml) for 3 h and subsequently ATP (5 mM) for 1 h. (a) The mRNA expression levels of caspase-1 were determined by RT-PCR analysis. (b) Caspase-1 mRNAs were quantified using real-time PCR. β-actin gene expression was used for normalization. The results are expressed as fold changes, considering one as the value of untreated cells. (c) The secretion of caspase-1 subunit p20 into the supernatants of THP-1 macrophages was assessed by ELISA. (d) Cell lysates and culture supernatants were Western-blotted to detect pro-caspase-1 p45 and caspase-1 subunit p20. Data are presented as means ± SE of three experiments preformed in duplicate. ^#^*P* < 0.01 versus untreated cells. *P < 0.01 versus HSM-untreated control (ethanol) cells. **^†^***P* < 0.05 versus HSM (1%) treated cells.

The cytokines IL-1β and IL-18 are generated as cytosolic precursors that require cleavage by the protease caspase-1 in order to generate biologically active cytokines. Caspase-1 itself is activated by several innate immune complexes termed inflammasomes^37^. To determine whether caspase-1 gene expression and activation are affected by the HSM ethanol extract, we pre-incubated THP-1 macrophages with HSM for 20 h prior to LPS and ATP treatments as mentioned above. As shown in Figure 4a and 4b, the HSM extract decreased caspase-1 mRNA expression in the activated macrophages treated with LPS and ATP. The HSM extract also significantly inhibited ATP-induced caspase-1 activation (secretion) in a dose-dependent manner (Figure 4c and 4d). These results suggest that the ability of HSM to decrease secretion of IL-1β and IL-18 is due at least in part to reduced caspase-1 gene expression and protein activation in the treated macrophages.

### HSM extract inhibits NLRP1 inflammasome expression and caspase-4 activation in ATP-treated macrophages

**Figure 5:**
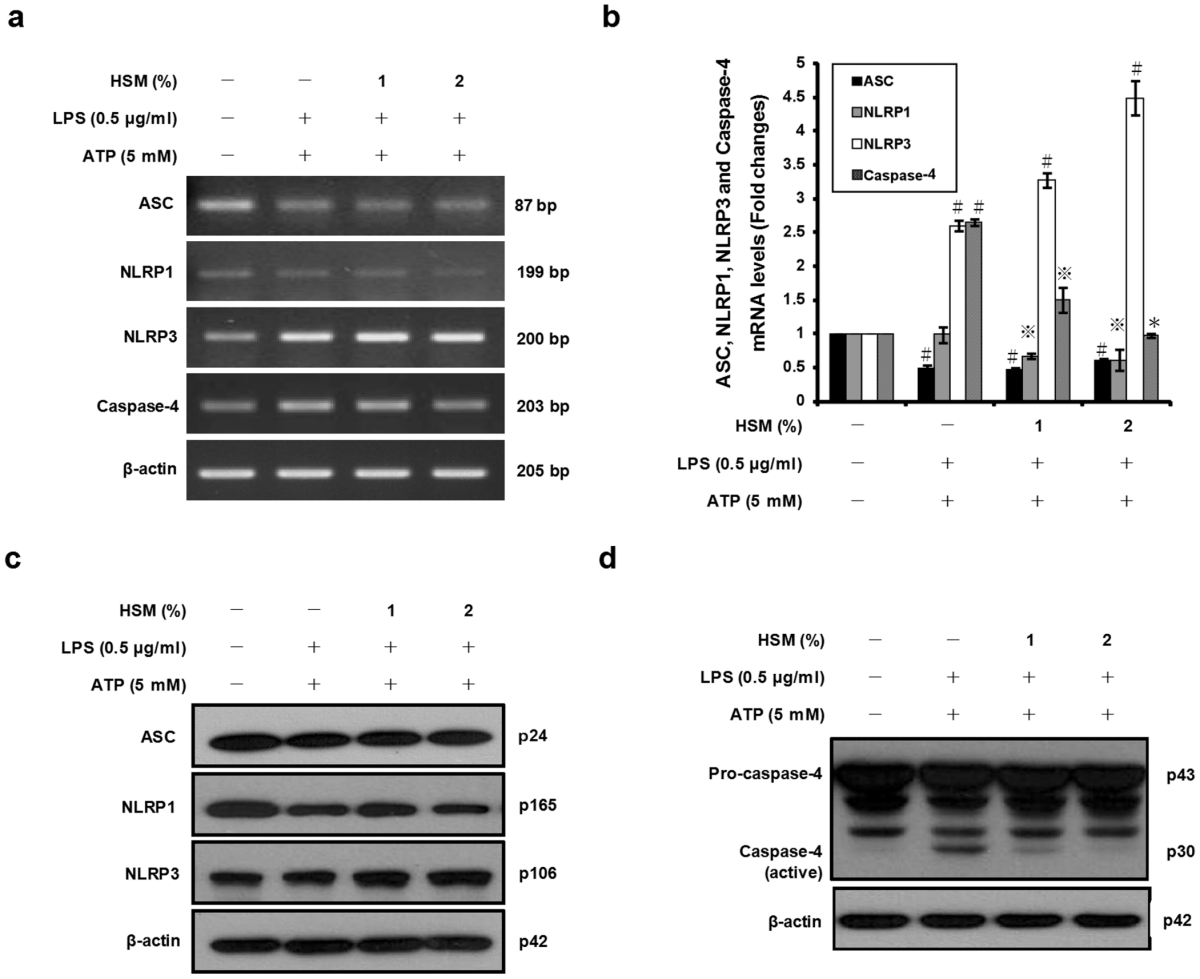
Effects of HSM on inflammasome components and caspase-4 activation in human macrophages. Cells were pretreated with various concentrations (1 or 2%) of HSM extract for 20 h, followed by treatment with LPS (0.5 μg/ml) for 3 h and ATP (5 mM) for 1 h. (a) The mRNA expression levels of ASC, NLRP1, NLRP3 and caspase-4 were determined by RT-PCR, using β-actin as the internal control. (b) ASC, NLRP1, NLRP3 and caspase-4 mRNAs were quantified using real-time PCR. β-actin gene expression was used for normalization. The results are expressed as fold changes, considering one as the value of untreated cells. (c) Cell lysates were analyzed by Western blot analysis using specific anti-ASC, anti-NLRP1 and anti-NLRP3 antibodies. (d) Cell lysates were analyzed for protein levels of caspase-4 by Western blot analysis. β-actin was used as an internal control. Data are presented as means ± SE of three experiments preformed in duplicate. ^#^*P* < 0.01 versus untreated cells. 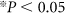 versus HSM-untreated control (ethanol) cells. *P < 0.01 versus HSM-untreated control (ethanol) cells.

Several inflammasome complexes, including the NLRP1 and NLRP3 inflammasomes, have been shown to activate caspase-1. Canonical inflammasomes are formed by three components that include an NLR family protein (e.g., NLRP1 or NLRP3), the adaptor protein ASC, and pro-caspase-1^58^. To examine whether the NLRP1 and NLRP3 inflammasomes may be involved in the anti-inflammatory activity of HSM, we measured the mRNA and protein expression levels of NLRP1, NLRP3, and ASC using RT-PCR, quantitative real-time PCR, and Western blot analyses. As shown in Figure 5a–c, pre-treatment of the cells with HSM decreased NLRP1 mRNA and protein levels compared to cells treated only with LPS and ATP. Conversely, the expression of NLRP3, which was clearly induced by LPS and ATP, was further increased in cells pretreated with HSM (Figure 5a–c). In addition, the level of ASC expression, which decreased following LPS and ATP treatments, appeared unchanged by HSM pre-treatment. These results suggest that the HSM extract may reduce IL-1β and IL-18 secretion by down-regulating NLRP1.

Recent studies have shown that pro-IL-1β and pro-IL-18 are also substrates of non-canonical inflammasomes containing the protease caspase-4, and that this proteolytic enzyme is able to generate the biologically active form of the cytokines^56^,^59^. To determine whether the HSM extract may affect caspase-4 gene expression in macrophages treated with LPS and ATP, we performed RT-PCR and quantitative real-time PCR assays using caspase-4 specific primers. As shown in Figure 5a and 5b, pre-treatment with HSM resulted in a significant decrease of caspase-4 gene expression in LPS-primed macrophages stimulated with ATP. Western blot analysis of HSM-treated cells also showed a significant reduction of active caspase-4 in cell lysates (Figure 5d). Taken together, these results suggest that the HSM-mediated reduction of IL-1β and IL-18 secretion by macrophages is due to both down-regulation of the NLRP1 inflammasome, and decreased activity of caspase-4.

### HSM extract down-regulates P2X_7_ receptor expression and ROS production in LPS-primed and ATP-stimulated macrophages

Previous studies have shown that activation of the purinergic receptor P2X_7_R by extracellular ATP is required for caspase-1 activation, a process that leads to processing and release of both caspase-1 and mature IL-1β into the culture medium of activated macrophages^60^,^61^,^62^.

**Figure 6:**
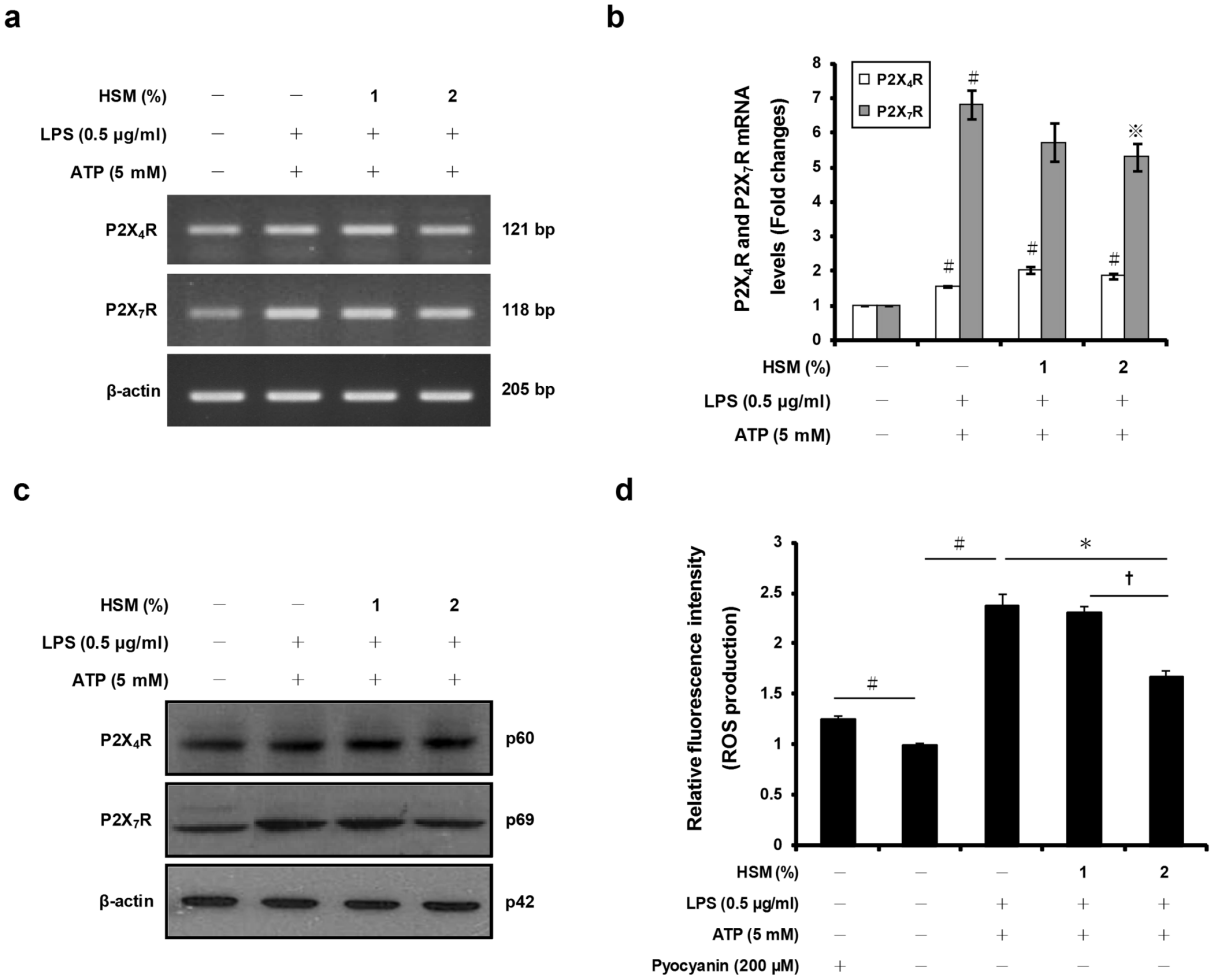
Effects of HSM on the expression of P2X_4_R and P2X_7_R and ROS production in human macrophages. Cells were pretreated with various concentrations (1 or 2%) of HSM extract for 20 h, followed by treatment with LPS (0.5 μg/ml) for 3 h and ATP (5 mM) for 1 h. (a) The mRNA expression levels of P2X_4_R and P2X_7_R were determined by RT-PCR, using β-actin as the internal control. (b) P2X_4_R and P2X_7_R mRNAs were quantified using real-time PCR. β-actin gene expression was used for normalization. The results are expressed as fold changes, considering one as the value of untreated cells. (c) Cell lysates were analyzed by Western blot analysis used specific anti-P2X_4_R and anti-P2X_7_R antibodies. (d) ROS production was measured with the total ROS detection kit, using a fluorescence microplate reader. Pyocyanin (200 μM), a ROS inducer, was used as a positive control for ROS formation. Data are presented as means ± SE of three experiments preformed in duplicate. ^#^*P* < 0.01 versus untreated cells. 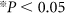 versus HSM-untreated control (ethanol) cells. *P < 0.01 versus HSM-untreated control (ethanol) cells. **^†^***P* < 0.05 versus HSM (1%) treated cells.

To determine whether the HSM extract may influence expression of P2X receptors (i.e., ATP-gated channels), we pre-treated THP-1 macrophages with HSM extract (1 or 2%), and then with LPS and ATP, prior to measuring the mRNA and protein expression levels of P2X_7_R and P2X_4_R (receptor for lower concentrations of ATP than P2X_7_R) using RT-PCR, quantitative real-time PCR, and Western blot analyses. As shown in Figure 6a–c, the up-regulation of P2X_7_R expression induced by ATP (and LPS) was suppressed in a dose-dependent manner by pre-treatment by the HSM extract. In comparison, the up-regulation of P2X_4_R expression induced by ATP was not affected by HSM treatment.

Previous studies have reported that activation of the P2X_7_R by ATP induces the production of ROS which are also required for activation of caspase-1 and secretion of IL-1β and IL-18^63^,^64^. To test whether the ROS production induced by ATP is also affected by HSM, we used a commercially-available detection kit to measure ROS production in macrophages that were pre-treated with HSM, prior to LPS priming and ATP activation. ATP treatment in LPS-primed macrophages caused a significant increase of ROS production compared with untreated cells (Figure 6d; pyocyanin was used as a positive control for ROS formation). The increase in ROS production could be suppressed by pre-treating macrophages with HSM extract (Figure 6d). Taken together, these results indicate that the HSM ethanol extract reduces IL-1β and IL-18 secretion in activated macrophages in part by down-regulating the ATP purinergic receptor P2X_7_R and reducing ATP-induced ROS production.

## Discussion

*C. sinensis* is a well-known traditional Chinese medicinal mushroom used for the treatment of a variety of human diseases such as liver disease, respiratory disease, renal dysfunction, heart disease, hyperglycemia, and hyperlipidaemia^6^,^7^. Recent studies have demonstrated that *C. sinensis* possesses immunomodulatory properties that, depending on the context, both activate and inhibit the immune system^12^,^65^,^66^. In the present study, we evaluated the effects of an HSM ethanol extract on the secretion of IL-1β and IL-18 induced by ATP in LPS-primed macrophages. We observed that pre-treatment with the HSM ethanol extract reduced the production of pro-inflammatory cytokines IL-1β and IL-18 in these cells. This finding is consistent with previous reports showing that *C. sinensis* extracts down-regulate the production of IL-1β in other LPS-activated immune cells^24^,^25^. However, our results also show that the HSM extract increases the transcription levels of the IL-1β and IL-18 precursors in LPS-primed and ATP-stimulated macrophages. Previous studies have reported that *Cordyceps militaris*, another Cordyceps species which is different from both natural *C. sinensis* and cultured HSM, induces IL-1β and IL-18 mRNA expression in murine RAW264.7 macrophages^67^,^68^. Our results indicate that the HSM extract suppresses the production of IL-1β and IL-18 through a mechanism other than inhibition of mRNA expression.

Since the inflammasomes are involved in IL-1β and IL-18 secretion, we investigated whether the reduced IL-1β and IL-18 secretion in HSM-treated cells was mediated by these molecular complexes. Assembly of the inflammasomes results in activation of the protease, caspase-1. Activated caspase-1 is responsible for processing of pro-IL-1β and pro-IL-18 and secretion of the mature cytokines^37^. Our results show that LPS-primed macrophages pre-incubated with the HSM extract causes decreased activation of caspase-1 in ATP-treated macrophages. Treatment with HSM also decreases caspase-1 mRNA expression.

Greten et al. demonstrated previously that activation of the nuclear transcription factor NF-κB induces pro-IL-1β mRNA synthesis and inhibits caspase-1 activation in macrophages^69^. In addition, NF-κB is activated in response to various inflammatory stimuli, including bacterial LPS, cytokines, and viral infection^70^. Based on these results, we suggest that the HSM extract may activate NF-κB and lead to induction of pro-inflammatory cytokines IL-1β and IL-18 precursors and to inhibition of capase-1 activation in macrophages treated with LPS and ATP.

Our mechanistic studies show that HSM-dependent reduction of IL-1β and IL-18 production is due to a specific down-regulation of the NLRP1 inflammasome and subsequent inhibition of caspase-1 activity. A recent study by Hsu et al. showed that MDP stimulation induces the association of NOD2 with NLRP1 to form a complex that activates caspase-1 and triggers processing and secretion of IL-1β in macrophages^71^. NLRP1 also plays a crucial role in *Bacillus anthracis*-induced IL-1β secretion^71^. Additionally, THP-1 monocytes that were differentiated into macrophages with phorbol 12-myristate 13-acetate (PMA) and further treated with LPS or MDP plus ATP induced NLRP1 inflammasome assembly, caspase-1 activation, and IL-1β secretion^52^,^53^.

To our knowledge, our study is the first report demonstrating the effects of HSM extract on an inflammasome in THP-1 macrophages activated with LPS and ATP. Furthermore, we make the unexpected observation that a mushroom used in traditional medicine can also activate a non-canonical inflammasome. However, the HSM ethanol extract studied here also increased NLRP3 mRNA and protein levels in the activated macrophages. Recent evidence indicates that NLRP3 expression is tightly controlled by the activation of NF-κB, and that NF-κB inhibition leads to a dose-dependent reduction of NLRP3 protein induced by LPS^72^. This finding further supports the possibility that the HSM extract may induce NF-κB activation, which also increases NLRP3 expression and induces accumulation of IL-1β and IL-18 precursors in LPS-primed and ATP-stimulated macrophages.

ATP-induced P2X_7_R activation promotes the production of ROS, which in turn stimulates activation of the NLRP3 inflammasome^63^. In this study, we demonstrated that pre-treatment of LPS-primed macrophages with HSM extract significantly inhibits ATP-induced P2X_7_R expression. Moreover, our results show that ATP-induced ROS production is suppressed by the HSM extract. In agreement with these findings, previous studies have shown that the *H. sinensis* preparation CorImmune displays antioxidant activity and protects tissues and cells against free radical-induced damage^73^,^74^. Anti-oxidant activity was also reported for natural *C. sinensis*, and this activity might be derived partly from the polysaccharide fraction of *C. sinensis* water extracts^75^. Recently, caspase-4 expression was shown to be required for caspase-1 activation and maturation of pro-IL-1β and pro-IL-18 in keratinocytes and activated THP-1 macrophages, suggesting that caspase-4 may act upstream of a non-canonical inflammasome^56^. Interestingly, production of ROS plays an important role in ER stress induction, which further leads to proteolytic cleavage of caspase-4^76^,^77^. To address whether caspase-4 expression and activation is also regulated by HSM, we examined the expression of caspase-4 in activated macrophages. Pre-treatment of the cells with HSM resulted in a significant reduction of caspase-4 expression and activation compared with control, untreated cells. These results indicate that HSM compounds may act upstream of the inflammasome and result in down-regulation of caspase-1 activation and reduced IL-1β and IL-18 secretion.

We are currently investigating the compounds responsible for producing the anti-inflammatory effects of HSM. The nucleoside derivative 3′-deoxyadenosine—also called “cordycepin”—has been described in the past as an active ingredient of *C. sinensis* extracts^78^,^79^,^80^. Studies have shown that synthetic cordycepin produces anti-inflammatory effects on cultured cells^81^,^82^,^83^. However, chemical analyses performed by other groups showed that, while cordycepin is found in *C. militaris*, this compound is usually absent in both natural *C. sinensis* fruiting bodies and cultured HSM^84^,^85^. In fact, our own high-performance liquid chromatography analysis confirmed that cordycepin is not detected in the HSM ethanol extract studied here (Y.-F. Ko, J. D. Young, unpublished observations). Our preliminary chemical analysis of the HSM ethanol extract indicates that the compounds responsible for the anti-inflammatory effects of HSM have molecular weights ranging from 400 to 1,500 Da, but that neither polysaccharides nor adenosine can be detected in the extract (Y.-F. Ko, J. D. Young, unpublished observations). Structural studies are in progress in our laboratories to identify the chemical nature of this anti-inflammatory activity.

**Figure 7:**
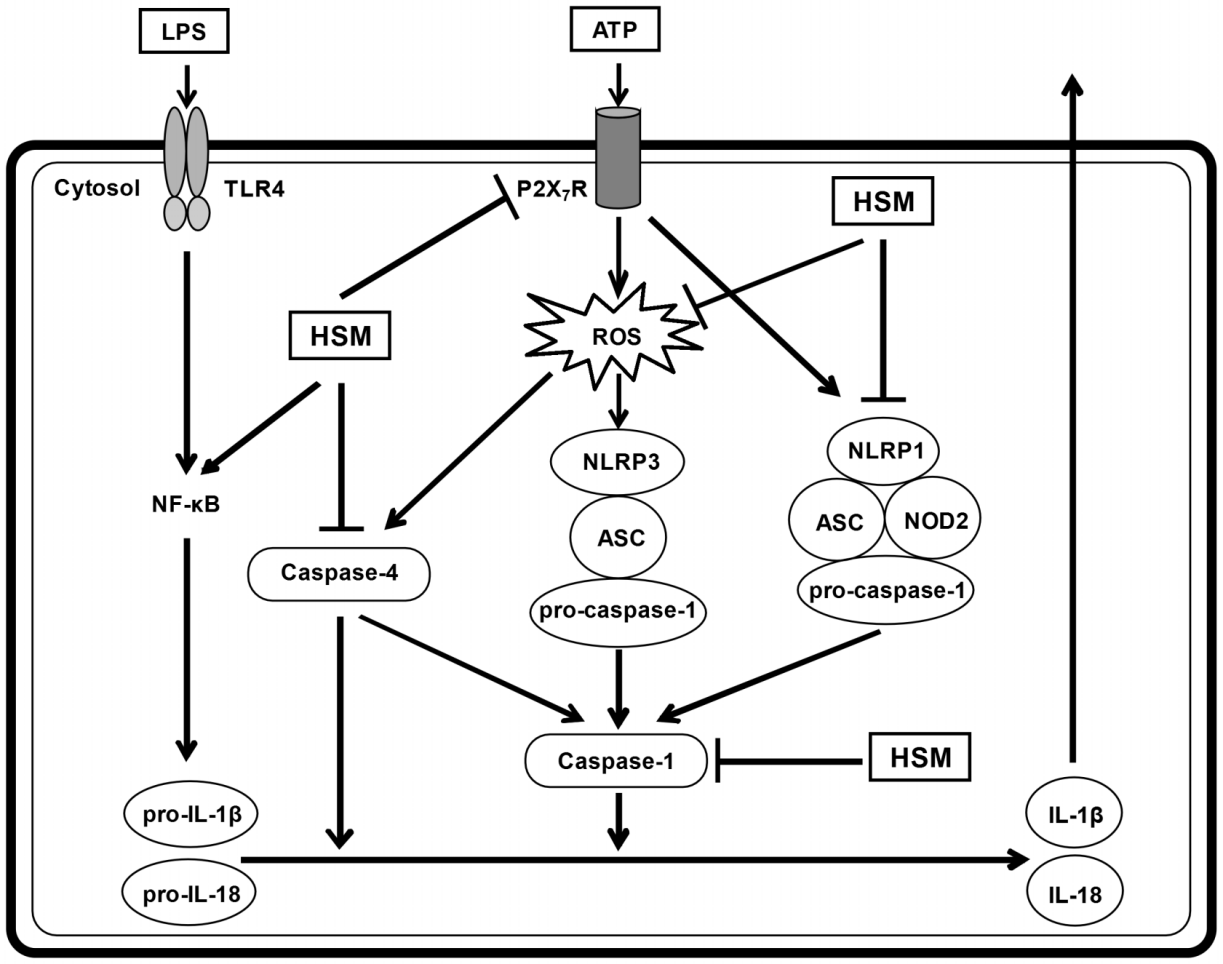
Schematic model for the reduction of IL-1β and IL-18 secretion in LPS-primed and ATP-stimulated macrophages treated with HSM. The HSM extract down-regulated P2X_7_R expression, ROS production, NLRP1 expression, and caspase-1 and caspase-4 activation, which together inhibited the secretion of IL-1β and IL-18. TLR4: Toll-like receptor 4.

In conclusion, our results demonstrate that the HSM extract is a potent inhibitor of ATP-induced caspase-1 activation and secretion of IL-1β and IL-18 in LPS-primed human macrophages. Figure 7 summarizes the intracellular pathways affected by the HSM extract. The reduction of IL-1β and IL-18 secretion by the HSM ethanol extract in activated macrophages is associated with inhibition of P2X_7_R expression, ROS production, NLRP1 expression, and caspase-1 and caspase-4 activation.

The cytokines IL-1β and IL-18 can induce inflammation, fever, and tissue damage in humans. Blocking secretion of these cytokines with HSM could represent a viable strategy to relieve symptoms associated with inflammatory disorders such as asthma, rheumatoid arthritis, inflammatory bowel disease, and other autoimmune diseases.

## Methods

### Chemicals and reagents

ATP, LPS and PMA were purchased from Sigma-Aldrich (St. Louis, MO). Cell culture medium (RPMI 1640), FBS, penicillin and streptomycin were purchased from Life Technologies (Grand Island, NY). For Western blot analysis, the antibodies against IL-1β and caspase-4 were obtained from Cell Signaling Technology (Beverly, MA); the ones against ASC, P2X_7_R, pro-IL-1β and IL-18 were from Santa Cruz Biotechnology (Santa Cruz, CA); and those against NLRP3 and P2X_4_R were from Sigma-Aldrich. The antibody directed against caspase-1 was purchased from Millipore (Billerica, MA); the one against NLRP1 was from Enzo Life Sciences (Farmingdale, NY); and the one against β-actin was from Novus Biologicals (Littleton, CO). The secondary antibodies used were horseradish peroxidase-conjugated anti-rabbit and anti-mouse IgGs (Santa Cruz Biotechnology).

### Fungal strain and preparation of the ethanol extract

The *H. sinensis* strain originally selected and characterized at Chang Gung Biotechnology (Taipei, Taiwan) was validated by comparison of its internal transcribed spacer DNA with that of natural *C. sinensis*^23^. The ethanol extract was prepared by adding 400 g of *H. sinensis* mycelium powder to 10 liters of 95% ethanol (v/v) into a Buchi R220 vacuum concentrator (Zurich, Switzerland), followed by stirring at a speed of 120 rpm for 60 min at 80°C. The HSM solution was cooled to room temperature and centrifuged at 4,500 rpm for 30 min at 4°C using a Sorvall RC 3C Plus centrifuge (Thermo Fisher Scientific, Waltham, MA). The supernatant was collected and concentrated to a final volume of 2 liters by using the Buchi R220 vacuum concentrator at 65°C. The HSM ethanol extract was finally sterilized by filtration through a 0.45 μm filter (Millipore), and stored at 4°C in dark glass bottles until use.

### Cell culture and treatments

Human acute monocytic leukemia THP-1 cells (American Type Culture Collection, TIB-202) were cultured in RPMI 1640 medium supplemented with 10% (v/v) heat-inactivated FBS, 100 units/ml of penicillin, and 100 μg/ml of streptomycin. THP-1 cells were incubated at 37°C in a cell culture incubator containing 5% CO_2_ and saturated humidity. The experiments were performed with cells plated in 6-well plates at 2 × 10^6^ cells per well. The cells were differentiated to adherent macrophages by overnight culture in complete medium supplemented with 500 ng/ml of PMA, and then with fresh complete medium for an additional 2 days. THP-1 macrophages were pre-treated for 20 h with 1 or 2% of HSM extract or with 2% ethanol as a control, followed by treatments with LPS (0.5 μg/ml) for 3 h and ATP (5 mM) for 1 h. Cell culture supernatants were harvested at 14,000 × *g* for 5 min at 4°C, and the supernatants were collected and stored at –80°C for cytokine assay. In addition, cell lysates were resuspended in lysis buffer for RNA extraction and Western blot analysis.

### MTT assay for cell viability

Cell viability was determined using a commercial MTT-based cytotoxicology test kit (Sigma-Aldrich), which detects viable cells colorimetrically based on the detection of the purple formazan compound produced by viable cells. THP-1 cells were initially seeded in 96-well plates (1 × 10^5^ cells/well) for 24 h. For macrophage differentiation, cells were treated and incubated with PMA as described above. Cell culture media were replaced by complete media containing different concentrations of HSM extract ranging from 1 to 5%, followed by incubation for 24 h. After incubation, 10 μl of MTT (5 mg/ml) were added to each well, and the plates were incubated at 37°C for 4 h. Each well was eluted and the precipitates were dissolved with 100 μl of MTT solubilization solution. Cell viability was obtained by calculating absorption values at 570 nm using a VersaMax microplate ELISA reader (Sunyvale, CA). All treated samples and controls were tested in triplicate.

### Enzyme linked immuno sorbent assay (ELISA)

THP-1 macrophages (2 × 10^6^ cells/well) in 6-well culture plates were pre-incubated with the HSM extract (1 or 2%) in 1 ml of complete medium for 20 h, followed by treatment with LPS (0.5 μg/ml) for 3 h and treatment with ATP (5 mM) for 1 h. Cell culture supernatants were collected and centrifuged at 10,000 × *g*, 4°C for 5 min to remove cell debris. Levels of secreted IL-1β, IL-18 and activated caspase-1 in cell culture supernatants were measured using commercially available ELISA kits (R&D Systems, Minneapolis, MN) according to the manufacturer's instructions.

### Measurement of ROS production

Total ROS/Superoxide detection kit (Enzo Life sciences) was used to assess ROS production in THP-1 macrophages. Briefly, cells were first seeded (1 × 10^5^ cells/well) in 96-well culture plates for 24 h. For macrophage differentiation, cells were treated and incubated with PMA in as described above. Cell media were replaced by complete media containing different concentrations of HSM extract (1 or 2%) and then incubated for 20 h, followed by treatment with LPS (0.5 μg/ml) for 3 h and treatment with ATP (5 mM) for 1 h. In addition, cells were treated with the ROS inducer pyocyanin (200 μM), as a positive control, for 30 min at 37°C. After treatment, cells were washed with 200 μl of 1× wash buffer and loaded with 100 μl of ROS/Superoxide detection reagents, and then incubated at 37°C for 1 h. The plates were read using a VersaMax microplate ELISA reader (Sunyvale, CA) at 520 nm after excitation at 488 nm. The increase in relative fluorescence intensity was used to determine intracellular ROS production.

### Protein extraction and western blot analysis

Cell extracts and cell culture supernatants were analyzed by Western blot analysis. Twenty hours after HSM treatment, cells were treated with LPS (0.5 μg/ml) for 3 h and subsequently with ATP (5 mM) for 1 h. The HSM-treated cells were washed twice with PBS and suspended in RIPA lysis buffer (50 mM Tris-HCl, pH 7.4, 150 mM NaCl, 0.25% deoxycholic acid, 1% Nonidet P-40, 1 mM EDTA) (Millipore) and complete protease inhibitor cocktail (Roche, Mannheim, Germany). Cell suspensions were incubated on ice for 30 min and centrifuged at 15,000 × *g* for 30 min at 4°C. The supernatants of cell suspensions were harvested as described above and stored at –80°C. Total protein concentration was determined using the Bio-Rad Bradford assay (Herculus, CA). Proteins were separated by electrophoresis in 8-to-12% SDS-polyacrylamide gels and transferred onto Millipore PVDF membranes. Specific proteins were detected using the appropriate primary and secondary antibodies before visualization using enhanced chemiluminescence detection kit (Millipore).

### RNA isolation and reverse transcriptase-polymerase chain reaction (RT-PCR) analysis

Total RNA was extracted from THP-1 cells using total RNA mini-kit according to the manufacturer's instructions (Geneaid, Taipei, Taiwan). Two μg of RNA were reversely transcribed in a reaction volume of 20 μl which contained an oligo (dT) primer, dNTP, and the SuperScript™ III reverse transcriptase (Invitrogen, Carlsbad, CA). The cDNA for ASC, caspase-1, caspase-4, IL-1β, IL-18, NLRP1, NLRP3, R2X_4_R, P2X_7_R and β-actin were amplified by PCR using the following specific primers: ASC forward primer 5′-ATCCAGGCCCCTCCTCAGT-3′, and reverse primer 5′-GTTTGTGACCCTCCGCGATAAG-3′; caspase-1 forward primer 5′-GAATGTCAAGCTTTGCTCCCTAGA-3′, and reverse primer 5′-AAGACGTGTGCGGCTTGACT-3′; caspase-4 forward primer 5′-GGTCATCATTGTCCAGGC-3′, and reverse primer 5′-CCATTGTGCTGTCTCTCC-3′; IL-1β forward primer 5′-AAAAGCTTGGTGATGTCTGG-3′, and reverse primer 5′-TTTCAACACGCAGGACAGG-3′; IL-18 forward primer 5′-GCTGAACCAGTAGAAGACAATTG-3′, and reverse primer 5′-ATCTGATTCCAGGTTTTCATCATCT-3′; NLRP1 forward primer 5′-ACCTGATCCCAAGTGACTGC-3′, and reverse primer 5′-TCTTCTCCAGGGCTTCGATA-3′; NLRP3 forward primer 5′-CTTCTCTGATGAGGCCCAAG-3′, and reverse primer 5′-GCAGCAAACTGGAAAGGAAG-3′; P2X_4_R forward primer 5′-GGATGTGGCGGATTATGTGATAC-3′, and reverse primer 5′-AGTGGTCGCATCTGGAATCTC-3′; P2X_7_R forward primer 5′-TGTGCCTACAGGTGCTACGCC-3′, and reverse primer 5′-GCCCTTCACTCTTCGGAAACTC-3′; and β-actin forward primer 5′-GAGACCTTCAACACCCCAGCC-3′, and reverse primer 5′-GGATCTTCATGAGGTAGTCAG-3′. Amplified PCR products were electrophoresed in a 2% agarose gel and visualized by ethidium bromide (Sigma-Aldrich) staining using a standard image system.

### Quantitative real-time PCR analysis

Quantitative real-time PCR was performed using LightCycler technology (Roche) with FastStart DNA Master^PLUS^ SYBR Green I (Roche) detection. Each LightCycler capillary was loaded with a total volume of 20 μl containing template cDNA, 250 nM sense and antisense primers, and 4 μl of 5× SYBR Green Master Mix. In all assays, cDNA was amplified using a standard program (10 min denaturing step; 50 amplification cycles of 10 s at 95°C, 10 s at 55°C, and 10 s at 72°C). Real-time PCR was performed with the same primer sequences stated above. Relative quantification of target gene expression was determined using a mathematical model described in the manufacturer's guidelines (Roche). Each PCR assay was performed in triplicate on two separate occasions for each experiment.

### Statistical analysis

Triplicate data for each experiment were presented as mean ± SE. Mean comparisons between HSM-treated and control untreated cells were analyzed using Student's *t*-test. *P* values below 0.05 were considered statistically significant.

## Author Contributions

T.-T.H., K.-Y.C., D.M.O., H.-C.L. and J.D.Y. conceived and designed the research. T.-T.H., Y.-H.W. and C.-Y.W. performed experiments. T.-T.H., Y.-H.W., Y.-F.K., C.-Y.W., J.M., C.-C.L. and H.-C.L. analyzed the data. T.-T.H., K.-Y.C., D.M.O., J.M., H.-C.L. and J.D.Y. wrote the manuscript.
